# EMPOWERMENT IN MOTION: ANALYZING THE EXPERIENCE OF WOMEN HIP-HOP DANCE CREW

**DOI:** 10.1093/geroni/igae098.2202

**Published:** 2024-12-31

**Authors:** Kyung Eun Lee, Hae Youn Min, Jinmoo Heo

**Affiliations:** Yonsei University, Seoul, Republic of Korea; Yonsei University, Seoul, Republic of Korea; Yonsei University, Seoul, Republic of Korea

## Abstract

Hip-hop dance is known as one of the dance genres, primarily associated with the younger generation. However, it has gained popularity among not only the youth but also the older generations worldwide. Participants in this study have formed hip-hop dance crews targeting middle and old-aged women, engaging in regular practices and performances to create and enjoy their leisure activities. According to the innovation theory (Nimrod & Kleiber 2007), individuals can achieve successful aging by actively engaging in leisure activities. This theory emphasizes the importance of continuously seeking new experiences and challenges, even in later stages of life. Therefore, this study aimed to explore the experiences of participation in the women’s dance crew based on the innovation theory. In-depth interviews were conducted with 16 middle and old-aged women regarding their motivations, identity as members, and outcomes. The interview results confirmed that participants initially began their activities for various reasons (e.g., family recommendations, maintaining health), but continued their activities due to intrinsic motivation. Interestingly, it was found that both self-preservation innovation (leisure activities related to previous life experiences) and self-reinvention innovation (completely new leisure activities) were simultaneously discovered among the participants. Furthermore, participants were able to enhance psychological, physical, and social well-being through dance activities. These findings align with the key factors proposed in the innovation theory. Consequently, it was observed that middle and old-aged women preserve their past identities and explore new aspects of themselves by enjoying hip-hop dance, highlighting the significance of leisure activities in later life.

